# The Bootstrap Model of Prebiotic Networks of Proteins and Nucleic Acids

**DOI:** 10.3390/life12050724

**Published:** 2022-05-12

**Authors:** Thomas Farquharson, Luca Agozzino, Ken Dill

**Affiliations:** 1 Department of Chemistry, Stony Brook University, Stony Brook, NY 11794, USA; thomas.farquharson@stonybrook.edu; 2 Laufer Center for Physical and Quantitative Biology, Stony Brook University, Stony Brook, NY 11794, USA; luca.agozzino@alumni.stonybrook.edu; 3 Department of Physics and Astronomy, Stony Brook University, Stony Brook, NY 11794, USA

**Keywords:** origins of life, DNA-protein networks, protocells

## Abstract

It is not known how life arose from prebiotic physical chemistry. How did fruitful cell-like associations emerge from the two polymer types—informational (nucleic acids, xNAs = DNA or RNA) and functional (proteins)? Our model shows how functional networks could bootstrap from random sequence-independent initial states. For proteins, we adopt the foldamer hypothesis: through persistent nonequilibrium prebiotic syntheses, short random peptides fold and catalyze the elongation of others. The xNAs enter through random binding to the peptides, and all chains can mutate. Chains grow inside colloids that split when they’re large, coupling faster growth speeds to bigger populations. Random and useless at first, these folding and binding events grow protein—xNA networks that resemble today’s protein–protein networks.

## 1. Introduction

How did life originate 3.5 billion years ago from the prebiotic world before it? This puzzle is made more challenging by entangling three mysterious complexities together: diverse functional molecules (mostly proteins), molecules that store information and memory (in xNAs, i.e., DNA and RNA) and encapsulation of biomolecules inside cells.

There have been speculations about *what came first?* like the chicken-and-egg problem. Did life start as an RNA world [1,2,3]? Or, did metabolic reactions precede the enzymes that could catalyze them [4,5]? Or, was encapsulation first in a “Lipid World” [6]?

On the one hand, a Something-Came-First World would certainly be a wonderful convenience for modelers, requiring the fewest assumptions and parameters, at least for that step of early origins. On the other hand, some form of cooperativity must have been crucial to the story of the origins of life. Furthermore, what is convenient for modelers is not necessarily what happened in reality. An alternative view is that biology originated through the co-origination of multiple molecule types together, such as RNA and proteins (along with small molecules) [7,8,9,10].

The attractiveness of the multi-molecule world lies in the fact that it does not require an explanatory mechanism for the evolution of another molecule type. Both informational and functional molecules exist and evolve concurrently. The importance and evidence of mutually fruitful interactions between RNA and proteins at life’s origin has been recently elucidated [11,12]. Cationic proto-peptides, synthesized under plausible prebiotic conditions, were shown to react directly with RNA to produce mutually stabilizing partnerships: proto-peptides had longer lifespans and RNA duplexes had enhanced thermal stability.

The modeling challenge we take up here is not to seek a simpler problem that avoids the multi-molecule complexity, but rather to confront the more complex challenge of assimilating all three components—function, information and encapsulation—into a single model. We posit a model in which peptides and xNAs are produced and elongated inside protocells. The synthesis of new chains is coupled to the protocell growth rate and protocells grow and split when they become large. The xNAs and peptides can interact. When these biomolecules form complexes that accelerate chain elongation, protocell growth accelerates, leading to increased populations. This process is self-sustaining and grows functional biochemical networks that further increase protocell growth rates.

The present model builds upon the foldamer hypothesis [13]. In short, the hypothesis outlines a dynamical mechanism describing two features: (a) the physical basis for how short chains became longer chains with specific sequences; and (b) a structural and plausible kinetic basis for a prebiotic autocatalytic system. Furthermore, the present modeling is itself characterized as an autocatalytic system. Biomolecules in the system are sustained by what is in the environment and each reaction is catalyzed by a biomolecule type produced inside of the system. Autocatalytic systems are known to be important for life’s origin and have been characterized and explored extensively [14]. More specifically, previous work has indicated that autocatalytic sets could spontaneously develop in an RNA–peptide world [15]. We remark here two principal properties which differentiate the present model from previously elucidated autocatalytic sets: (a) catalysis, via the HP foldcat mechanism [13], has a basis in molecular structure and physical chemistry; and (b) the resulting chemical networks that form have topological properties with dependencies like those in today’s biology.

## 2. The Background and the Model

### 2.1. The Premises and Assumptions

We describe a speculative mechanism for how chain elongations of proteins and xNAs inside protocells otherwise acting stochastically could bootstrap prebiotic chemistry to grow and sustain increasingly complex interaction networks. The premises are found below:

- **A nonequilibrium driver.** Because life is now, and always must have been, out of equilibrium, we are at liberty to suppose some persistent nonequilibrium drive was present. There are many potential sources. Here, we assume the availability of amino acids and nucleic acids, and that both are persistently being polymerized. At first, these would produce only short-chain random sequences of xNAs or peptides. Such plausible syntheses have previously been demonstrated [16,17,18,19];
- **A propagation principle.** Today, life as a whole sustains, and never dies out, due to the survival-of-the-fittest propagation principle. Moreover, it is resourceful, creative and innovative, due to its ability to search and choose by mutation and selection. Without it, there is no biology. It results because changes in biomolecules lead to changes in cell growth rates, which lead to changes in cell populations. Here, we assume a simple physical precursor dynamic. We assume peptides and xNAs are encapsulated and polymerize inside colloids or vesicles, causing such protocells to grow and to divide by known surface-to-volume effects [20,21,22,23]. We assume, as others have done, that the amino acid and nucleic acid monomers from the surroundings can pass freely into the protocells, but that the chains inside are too long to pass back out [24,25,26];
- **Funneling in the molecule space**. We believe life originated more as a disorder-to-order process, and less as specific sequence actions or specific binding actions or specific recognition between polymers (such as the genetic code). Rather, we believe such specificity must have emerged from the propagation mechanism (see above) acting on random molecules.

Two of the premises, the propagation principle and funneling in the molecule space, bear a striking resemblance to the idea of reciprocally coupled XNOR gating which allows one to filter and link emergent life properties by interchanging antecedents and consequents in a “strange loop” [27].

A key property of both proteins and xNA molecules is simply the lengths and growth rates of their chains. We assume that nucleic acids polymerize to have chain length distributions that resemble most known polymerization processes [13,28,29,30], while peptides polymerize into differently shaped chain length distributions by virtue of their ability to collapse in water into compact—sometimes uniquely folded—structures and have sequence-dependent abilities for functionality. We accept that peptides are hydrophobic–polar (HP) polymers given by the previously elucidated foldamer hypothesis [13,31].

We note here that the difference whereby proteins are functional and xNAs are informational must have come early in the origins process; living systems need both functional and informational biomolecules. Proteins can fold so sequence determines structure. In contrast, xNAs are relatively stiff and rigid, so their properties are relatively independent of their sequences. The main distinction here is that functional biomolecule types would have needed to have a strong sequence–structure–function interdependence, whereas the informational biomolecule types required a strong *independence* of sequence to structure and function. Memory storage must be able to store any sequence without bias, so that all sequences, in principle, could be searched and sampled by mutation. To that point, functional biomolecules would therefore make for poor information storage units because different sequences have different physical properties, thus biasing which sequences would be searched and sampled by mutation.

Our present model recognizes the above distinction between molecule types. Foldability is a property of peptides and proteins. Furthermore, while some xNAs chains can also fold, they are stiffer chains, so they fold less frequently and without the sequence–structure relationship. However, we acknowledge that certain xNA sequences can fold, especially RNA sequences, and are capable of functional activity (i.e., ribozymes). However, at the current level of granularity of the present model, we do not expect the presence of ribozymes to dominate or change the observed conclusions. Ribozymes play a small role in contemporary biology; thus, we suppose they play a role similar in magnitude here.

### 2.2. The Growth and Split Mechanism

**Figure 1:**
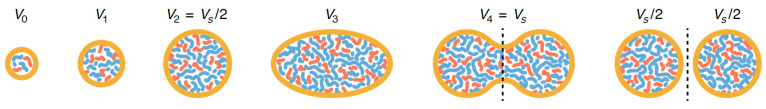
Protocells grow (and split) through chain elongation of its polymer inside. (Blue) Peptides as functional molecules. (Red) Nucleic acids as informational polymers. They grow in mass and length through synthesis. (Orange) The surface-to-volume ratio of the protocell decreases as new polymer chains are elongated inside. Subsequently, the protocell splits to produce two “daughter” protocells.

The main property that we require here is the length distributions of xNA chains and HP peptides. We consider the synthesis rate of a peptide of length *L*: If *k*(*L*) is the rate at which a molecule of length *L* is produced it is therefore simply inversely proportional to the length itself. If *k_x_* is the rate of elongation for polymers of type *X*, we have *k*(*L*) = *k_x_*/*L*; this is simply obtained by assuming a constant time interval between each monomer addition. Now we consider two different elongation rates for the informational molecule, *k_I_*, and for the functional molecules, *k_F_*. We can now write the rate equations for the two types of molecules as:

(1) $$\frac{dm_{\mu }}{dt}=\frac{k_{I}}{L_{\mu }},$$

(2) $$\frac{dn_{j}}{dt}=\frac{k_{F}}{L_{j}},$$

where $m_{\mu }$ and $n_{j}$ are the copy numbers for each different type of NA chain of type μ and HP chain of type j, respectively. Here, and in the rest of this paper, we will use Greek indices for *I*-type molecules and Latin indices for *F*-type ones. So $n_{j}\left(t\right)$ represents the number of functional molecules inside the droplet of type j, hence with length $L_{j}$, at time *t*, and similarly for $m_{\mu }\left(t\right)$. In this mechanism, the sole factor that determines the growth rates of the vesicle/droplet/protocell is simply how fast the nucleic acids and amino acid chains are elongating inside it. A visual schematic for protocell growth and splitting, due to chain elongation, is shown in Figure 1.

In water, nonpolar matter forms droplets due to the oil–water forces [32,33]. When droplets grow big, they split into two. We define $V_{s}$ to be the average droplet volume at the splitting point. We define $T_{s}$ to be the average time required to reach the splitting volume. Now, taking $v_{X}$ to be the volume increase due to the addition of a monomer to the molecule type *X*, and *N* and *M* to be the total number of different types of peptides and xNAs, respectively, we get:

(3) $$V_{s}=\sum_{j=1}^{N}n_{j}(T_{s})v_{F}L_{j}+\sum_{\mu =1}^{M}m_{\mu }(T_{s})v_{I}L_{\mu }.$$

The time scale for the elongation of a given molecule is faster than the protocell growth rate. Therefore, we assume that molecules which are incompletely synthesized (i.e., molecules shorter than the mature length of either $L_{j}$ and $L_{\mu }$) do not have an impact on the overall growth. Now we can solve Equations (1) and (2); and place them into Equation (3):

(4) $$n_{j}\left(t\right)=n_{j}^{0}+\frac{k_{F}}{L_{j}}t,$$

(5) $$m_{\mu }\left(t\right)=m_{\mu }^{0}+\frac{k_{I}}{L_{\mu }}t,$$

where $n_{j}^{0}\equiv n_{j}\left(0\right)$ and $m_{\mu }^{0}\equiv m_{\mu }\left(0\right)$. Now we take the intial time to be one of the splitting events, when the volume of the droplet is exactly $V_{0}=V_{s}/2$, and calculate the time at which the total population is sufficient to double this volume. Therefore, we have:

(6) $$2V_{0}=V_{0}+\left(v_{F}k_{F}N+v_{I}k_{I}M\right)T_{s},$$

where $V_{0}=\sum_{j=1}^{N}n_{j}^{0}v_{F}L_{j}+\sum_{\mu =1}^{M}m_{\mu }^{0}v_{I}L_{\mu }$ is the reference volume; it is the volume of the protocell right after a splitting event. From this expression we can calculate what effectively is the growth rate of a protocell with a given composition of initial populations $n_{j}^{0}$ and $m_{\mu }^{0}$ as the inverse of the splitting time:

(7) $$r_{0}=\frac{1}{T_{s}}=\frac{v_{F}k_{F}N+v_{I}k_{I}M}{\sum_{j=1}^{N}n_{j}^{0}v_{F}L_{j}+\sum_{\mu =1}^{M}m_{\mu }^{0}v_{I}L_{\mu }},$$

This is the reference growth rate for a system of protocells whose growing mechanism is controlled solely by chain elongation. Parameters used to calculate the reference growth rate are found in Table A1, Equations (A9)–(A11).

### 2.3. Intermolecular Interactions Drive Network Formation

In this model, proteins and xNA molecules in water interact through hydrophobic/polar interactions. xNAs are known to act not only by hydrogen bonding base pairing, but also by hydrophobic base stacking [34]. Here, when a hydrophobic chain monomer is exposed to water, it attracts other exposed hydrophobic monomers from either type of chain. We note that this binary interaction is just a simplification for the present modeling. Since many of the 20 amino acids were likely present at the origin of life, the full complexity of catalytic activities and binding interactions could have begun early. For our simple model here, our HP coding is a stand-in for how these early simple polymers could fold, recognize, bind, catalyze and react with one another.

Through this interaction mechanism, some random chains will associate with each other. This means that interaction networks can form. Here, we call this a protein–informational interaction (PII) network: every node represents one of the two molecule types. A link between two nodes corresponds to some form of interaction between the two corresponding molecules. A link can exist both between molecules of the same type (i.e., i−j or μ−ν for protein–protein and information–information molecules, respectively) or between molecules of different type (i.e., j−μ for protein–information interactions). Interactions can be of various types. While one outcome is aggregation (nonspecific interactions), another outcome is protein machines that have some primitive functional activity analogous to more contemporary enzymatic functions. The focus of this study is to explore a primitive form of functional interaction, and aggregation is only treated as an average.

**Figure 2:**
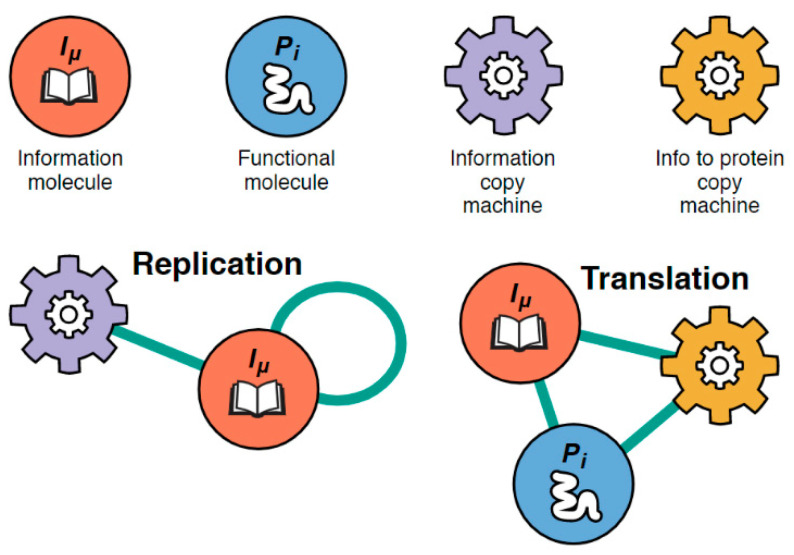
Defining the symbols representing the model’s PII nodes and important subgraphs. (Top row, left to right) Four major types of molecules exist in the PII interaction network: xNA molecules, protein molecules, ribosome-like proteins, and polymerase-like proteins. μ and i are iterators used to further distinguish between the different types of xNAs and proteins in the network (i.e., molecules which differ in sequence structure). Nodes represent the entire molecular population of a given type of molecule. (Bottom row, left to right) The two important types of subgraphs which denote primitive replicative function in the model: xNA copier subgraph and protein copier subgraph (see text).

Here we single out for special focus those proteins that are information copy machines (polymerase-like proteins) and those that are information-to-protein copy machines (ribosome-like proteins). We call them xNA copiers and protein copiers, respectively. Visual representations of the network’s nodes and subgraphs can be seen in Figure 2. Protein copiers are 3-molecule subgraphs; a protein that reads an xNA and produces another protein. xNA copiers are 2-molecule subgraphs; a protein that approximately duplicates an *I*-molecule. When such machines are catalytically active inside the protocell, they can increase its growth rate. Copy machines are considered “catalytically active” when the relevant completed replication or translation subgraph has been formed in the PII network. The discovery of new interactions is a consequence of changes in the sequence structure of a molecule type during foldamer-catalyzed elongation. We refer to these changes as “mutations.” Below we describe the term on a granular level and then show how it is represented in the present coarse-grain model.

In this present model, mutations are a consequence of life’s origin lacking specific binding actions or specific molecular recognition. We accept the foldamer hypothesis which posits that foldamers in autocatalytic sets can cross-catalyze foldamers of a different molecular sequence or can catalyze the elongation of a foldamer of the same sequence [13]. However, without molecular specificity, these elongation processes could have resulted in foldamer variants being synthesized. If we consider the following scenario: (a) foldamers of type *A* are responsible for catalyzing the elongation of a protein/xNA molecule of type *B*; (b) a mutation in the molecular space causes the synthesis of foldamers of type *A* to be polymerized as variants, *A^V^*; (c) foldamers of type *A^V^* now catalyze a new variant of protein/xNA molecule of type *B*, hence *B^V^*; and (d) *B*-type molecules are no longer synthesized, the population now reflects molecules of type *B^V^*; we can see how mutations provide new sequence variations to molecule types existing in the PII network.

The consequence of the mutation is what is simulated in the present model. When new molecular variants are synthesized, they will either gain or lose an interaction with a pre-existing molecule type accounted for in the model. The mutational effects are subtle; only a single interaction can be lost or gained at a time, per a single mutation. Mutations were random events. A randomly selected $A_{ij}$ matrix element was selected and changed to its opposite value: 1 → 0 or 0 → 1. Additionally, we did not track which of the two molecule types was the new variant, simply we simulate whether the mutation led to the loss or discovery of a molecular interaction between the two molecule types.

Most of the copy machines within the molecular population will not be functional; we represent this by an effectiveness parameter (α and β below). Effectiveness represents the catalytic accuracy of a population of a given copy machine type. It is assigned via a randomized process; an integer between 0 and 1 is chosen at random and assigned to a machine type. Larger effectiveness values (i.e., close to 1) indicate that the copy machines are efficient in synthesizing new polymers with little error in the sequence. Lower effectiveness values (close to 0) indicate that copy machines were error prone; only a few machines produce polymers having the intended sequence structure. When machines are effective, they can boost the production of polymers. When an *F*-molecule is interacting with a protein copier, its rate of production is subject to the effectiveness parameter. So, now the chain elongation rate is:

(8) $$\frac{dn_{j}}{dt}=\frac{k_{F}}{L_{j}}+\alpha \sum_{k\mu }A_{jk}A_{k\mu }n_{j}n_{k}m_{\mu }\delta (L_{j},L_{\mu }),$$

where α is a parameter that measures how effective the protein copier is, and $A_{jk}$ and $A_{k\mu }$ are the elements of the adjacency matrix of the network. These elements are 1 if there is a link between the two index nodes or 0 otherwise, and the Kronecker delta function only enforces that the transfer of information is possible if the lengths are the same. Similarly, when an xNA copier interacts with an *I*-molecule the elongation rate is given by:

(9) $$\frac{dm_{\mu }}{dt}=\frac{k_{I}}{L_{\mu }}+\beta \sum_{k}A_{\mu \mu }A_{\mu k}n_{k}m_{\mu }^{2},$$

where β is the effectiveness parameter of the xNA copier.

#### Protein Copiers as Peptides

We remark here that the protein copy machines in the present model are peptides, which goes against the known structure of contemporary ribosomes [35] and the supposed structures of primordial ribosomes (primarily RNA) [36,37]. We make three points here to justify our treatment of protein copy machines below:

- The RNA fraction in contemporary ribosomes ranges from 1/3rd–2/3rd in different organisms [38,39], indicating that the requirement of RNA as part of the machine is not a strong constraint;
- Contemporary organisms are known to have some nonribosomal peptide syntheses which are facilitated by other protein structures (i.e., nonribosomal peptide synthetase) [40]. As far as we know, there are no known equivalents for xNAs being duplicated by solely other xNAs;
- At the current level of coarseness of the present modeling, we simply approximate ribosomes as being catalytic elongators. Therefore, the network structure would not differ much from the observed results.

### 2.4. Computing the Growth Dynamics

Now, with these growth laws, we can determine the time the protocell would take to reach its splitting volume. These coupled differential equations can be solved numerically. However, it is possible to solve in the case of a single graph of the types in Figure 2 and then extrapolate the results in the case of many graphs of such type. In Equation (A5) we show that the overall protocell growth rate can be written as:

(10) $$r\approx r_{0}+\Delta r,$$

where Δr is a function of the topology of the interaction network. The process we now model is that of networks that change through mutations of the molecular sequences, leading to appearances or disappearances of links in the PII network. Mutations are random and can occur either in peptides or xNAs.

### 2.5. Mutations Drive the Network to Discover New Functional Relations, Affecting the Protocell’s Growth Rate

When the consequences of a mutation are modeled through the PII network (i.e., the appearance or loss of an interaction), the system’s discovery of a new interaction can lead to an increase in the growth rate of the protocell. Specifically, if the mutation leads to the formation of the appropriate subgraph necessary to represent xNA or protein copier function, the growth rate of the mutant-type protocell increases. Mutant-type protocells which discover these copy machine functions have enhanced growth rates. The new growth rate of the mutant is given by:

(11) $$r\approx r_{0}+\Delta r_{R}+\Delta r_{C},$$

where $\Delta r_{R}$ is the change in the growth rate due to the introduction of a protein copier, whereas $\Delta r_{C}$ is that due to the introduction of a xNA copier. Details of their expressions are given in Appendix A Equations (A1)–(A6). The growth rate r represents the growth rate of the protocell system where foldamer, xNA copier and protein copier catalysis all contribute to polymer elongation.

### 2.6. Polymer Aggregation Decrease Proto-Cellular Growth Rate

Polymer aggregation is a consequence of promiscuous interactions. When polymers aggregate, we predict there is a decrease in the growth rate. Aggregation removes polymers which are participating in replication or translation subgraphs and those used as templates in foldamer catalyzed elongation reactions. Consequently, the growth rate contributions from foldamer catalysis, protein copiers and xNA copiers would be less than their idealized calculated values. To reflect this feature, we include an aggregation cost:

(12) $$r=(r_{0}+\Delta r_{R}+\Delta r_{C})-\sum_{k}g(k),$$

where *g*(*k*) is the aggregation cost for each polymer type summed across all polymer types k in the model. The aggregation cost is given by:

(13) $$g\left(k\right)=\{\begin{array}{cc} 0, & d\left(k\right)<D\\ \delta \;d\left(k\right), & d\left(k\right)\geq \;D \end{array}\;,$$

where the aggregation cost for a polymer type is zero if its number of links d(k) is less than the threshold aggregation parameter, *D*; or its aggregation cost is given by δ d(k) if the number of links to the polymer type exceeds or equals the threshold parameter. δ is a static scaling parameter used to calibrate the aggregation cost to the magnitude of the growth rate. Parameters were set to *D* = 5 and δ = 0.005 to reflect the network size used and the magnitude of the reference growth rate.

### 2.7. Mutations Can Be Advantageous or Noise

The present model predicts that protocell populations evolve through two mechanisms which resemble natural selection and genetic drift: some changes in the distribution of polymers in a protocell have a relevant effect on the overall duplication rate, resulting in protocells with a higher chance to become common in a population; other changes have minimal to no effect, increasing the diversity of polymers distribution.

When an individual protocell, existing in a system of protocells, undergoes a mutation, there is a change in the sequence of one of the polymer types in its interaction network. There are three possible outcomes. First, if the mutation results in the network discovering some activity—the completion of either a xNA copier or protein copier subgraph—the cell growth rate and fitness increase. As the protocell population grows and reproduces, lineages from the mutant protocell have greater reproductive success than wild-type protocells. Consequently, each generation of protocells progressively look more and more like the mutant than the wild-type. In this way, beneficial mutations ultimately become fixed within the population. Second, other mutations can be deleterious, decreasing the growth rates of those protocells. Or, third, a mutation can be neutral, having no effect.

### 2.8. Mutations of the Individual Cells Propagate through the Population

In order to determine how likely a new mutation is to be selected by evolution, hence fixed in the population, it is necessary to consider a model of natural selection. The probability of fixation in such cases can be assumed to be given by Motoo Kimura’s expression [41], which simply expresses the probability that a given mutation with some selective advantage will ultimately be present in the entire population:

(14) $$\mu =\frac{1-e^{-2s}}{1-e^{-4Ns}},$$

(15) $$s=\log r_{\mathrm{mut}}-\log r_{\mathrm{wt}},$$

where s is the change in fitness due to a mutation, assuming that fitness is given by the log of the growth rate. *N* is the size of the protocell system and a parameter in the simulation. Simulated evolution trajectories used *N* = 100,000 protocells. Neutral mutations are fixed with a probability of [41]:

(16) $$\mu =\frac{1}{2N}.$$

### 2.9. Computer Simulations of the Model

**Figure 3:**
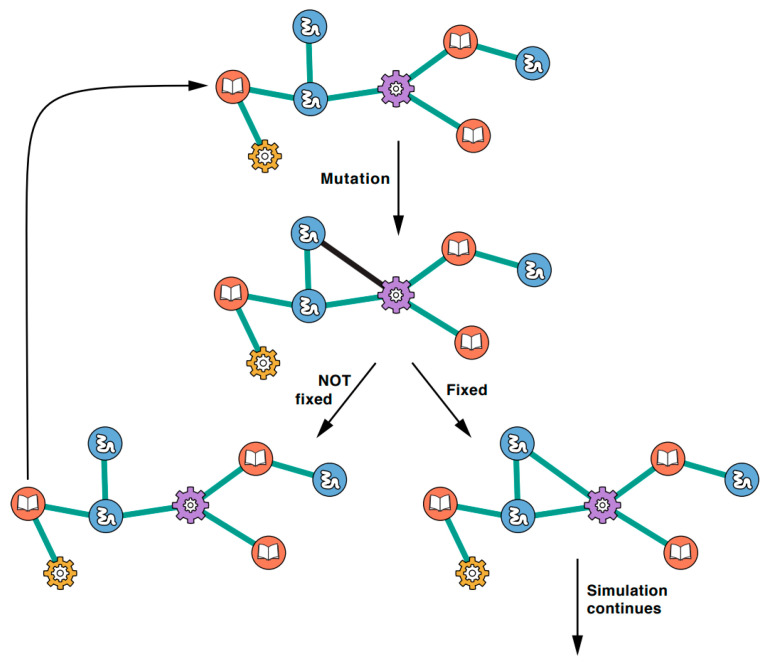
The available actions at each time step. (Top) A mutation can add a link (black line) or remove one. (Middle) That link either becomes fixed into the network (bottom right, green link) or not fixed (bottom left). The network now proceeds to the next time step.

The initial wild-type PII was a randomly generated $A_{ij}$ symmetric adjacency matrix with a size given by *N* + *M*. Network sparsity was determined by an adjustable probability whereby a given $A_{ij}$ matrix element is assigned a zero or a one. The adjacency matrix is mapped into the corresponding adjacency graph, which is the first wild-type PII interaction network for a protocell in a system of identical wild-type protocells; see Figure 3. The nodes on that graph represent different types of functional polymers (blue), informational polymers (red), a xNA copier (purple) and a protein copier (yellow). Each type of polymer has an initial population size and an assigned length. A link (edge) in the graph indicates an intermolecular interaction between polymers of either the same type (self-loop) or different types.

The growth rate of a protocell with the given wild-type PII network was calculated using Equation (7). The first mutation is then introduced into the system and a link/interaction is either lost or discovered. Here the growth rate of a protocell with the mutant PII network is calculated using Equation (12). The log of the growth rate for both the wild-type network and mutant network are taken and further evaluated using Equation (15) to give the selective advantage, *s*. If s=0, then the fixation of the mutation in the population is driven by genetic drift. If s≠0, fixation is driven by natural selection. If it is not fixed by either evolution force, then the wild-type network “wins out” over the mutant network (left path in Figure 3). The mutant protocell’s mutation falls out of the population after multiple generations of growth and selection with the wild-type PII network being the only available alternative. The process then repeats again with the wild-type network. If the mutation is fixed by either evolution force, then the mutant network becomes the dominant network-type in the population (right path in Figure 3) after multiple generations of growth and selection. The previous wild-type network is lost, and the mutant becomes the wild-type network. The process then repeats until a preset number of mutations have been introduced. Seven evolution trajectories were simulated for a protocell system containing 100,000 individuals. For each simulation 1,000,000 mutations were introduced into the system and the simulation ended at the 1,000,000th mutation.

## 3. Results and Discussion

### 3.1. When a Network Discovers Complete Copier Subgraphs, Its Protocell Grows Faster

**Figure 4:**
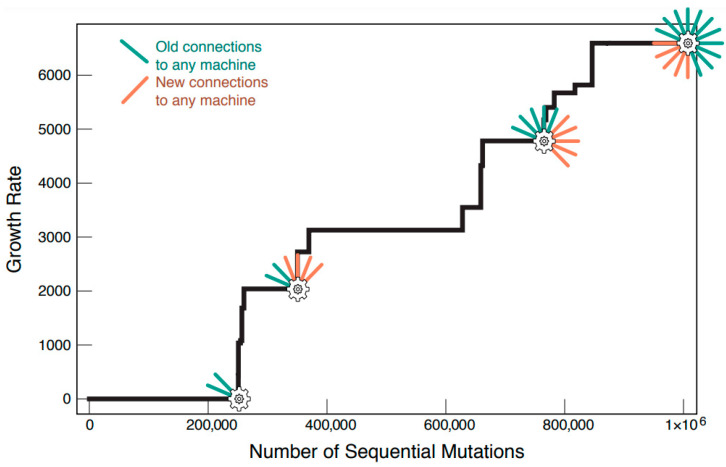
A protocell’s growth rate ratchets up over time as random mutations in the network happen to discover and lock in the specific interactions that specify the subnet machines in Figure 2.

When the above processes are modeled, the model predicts survival-of-the-fittest behavior. Figure 4 shows a time graph simulating introduced mutations in a protocell population. When the network of a mutant protocell discovers a beneficial mutation (i.e., the discovery of either complete copy machine subgraph), it wins out against the alternative wild-type in the population. This feature is highlighted by the ever-increasing growth rate of the population. Protocell generations resemble parents that had discovered additional ways to elongate their polymer chains. We can relate this to a simple tournament bracket analogy. When we compare the growth rate of a wild-type and mutant-type protocell, the one with the higher growth rate will be nonrandomly selected for and its lineage continues onward to become the new wild-type. Another mutation arises in the population and the new mutant and wild-type are compared, with nonrandom selection once again favoring the protocell with the higher growth rate. This cycle repeats with the result being a maximization of proto-cellular fitness.

We note here that periods of no change in the growth rate do no imply stagnation in the evolution of the protocell population. Mutants with neutral mutations can still win out over the wild-type variant, but this due simply to chance. Given the network size chosen in our simulations, on average, <0.005% of all mutations out of 1,000,000 were beneficial. The remaining 95.995% of mutations were neutral. The frequency by which neutral mutations occurred does not diminish their importance. Neutral mutations are important for discovering the requisite interactions for completion of either copy machine subgraph.

As a general principle of the model, novel protein copier links are harder to discover and subsequently fix compared to links discovered for the xNA copier. The reason is two-fold: the subgraph depiction for protein copier function in this model requires one additional link than that of xNA copier function (Figure 2), and there is a length requirement for primitive translation between the functional molecule and the informational molecule. To maintain simplicity in the model we have assumed that the earliest form of the genetic code had a one-to-one correspondence between an amino acid and a nucleic acid. In essence, the functional molecule and the informational molecule in the translation subgraph must be the same length. The addition of the length requirement increases the time it takes for the first translation subgraphs to appear in the network. However, once a few of them have been established, subsequent interactions among participating functional and informational molecules in the established subgraphs become more facile.

**Figure 5:**
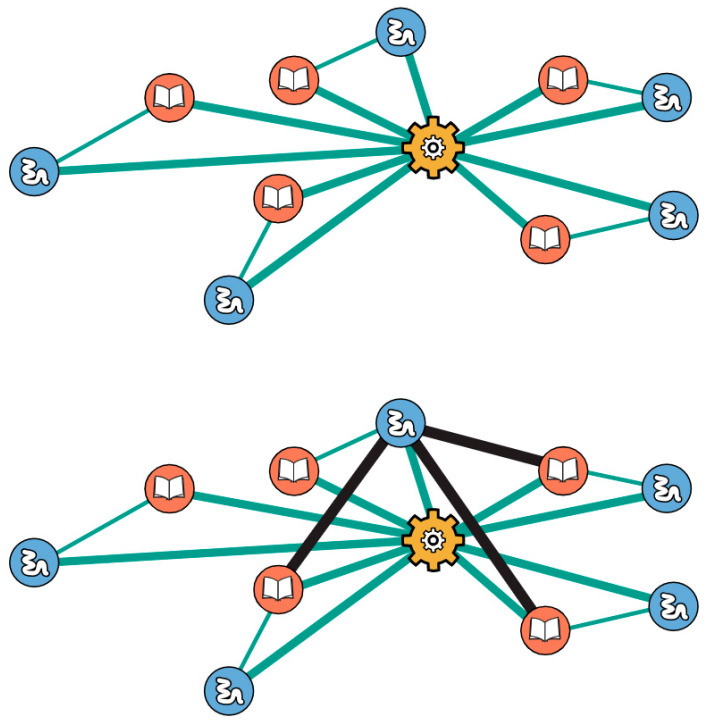
Cooperativity: when a network has at least two protein copier subgraphs, subsequent formation of more becomes more facile; see text.

Another key feature of the bootstrap model is that it simulates a form of cooperativity that is known to occur in today’s cellular protein–protein interaction (PPI) networks [42]. In short, bigger subgraphs in PPI networks have higher probability of forming an added link than smaller subgraphs have. In the present model, this applies to the two types of subgraphs: the 2-link xNA copier (transcription) and the 3-link protein copier (translation). When a 2-link translation or 1-link transcription subgraph is present, the probability that the subgraphs will grow into their respective 3-link and 2-link subgraphs is enhanced. An interaction which completes a copier subgraph brings with it an increase in growth rate. Copier subgraphs which have some, but not all, of the requisite interactions for completion bootstrap the formation and fixation of the remaining interaction(s) which complete it. This form of cooperativity is exemplified with the protein copier. When a few of the 3-link translation subgraphs have already been discovered, the subsequent discovery for more is enhanced. Consider the case shown in Figure 5.

Functional and informational molecules participating in complete subgraphs have pre-existing interactions with the protein copier. A new interaction which arises between a functional and informational polymer on two different 3-link subgraphs can lead to the immediate formation of another 3-link translation subgraph. Consequently, there is an increase in growth rate. This observed cooperativity suggests that interactions between functional and informational polymers in different protein copier subgraphs are more facile than similar interactions elsewhere in the network.

### 3.2. Networks Evolve to Become Bigger and More Complex

**Figure 6:**
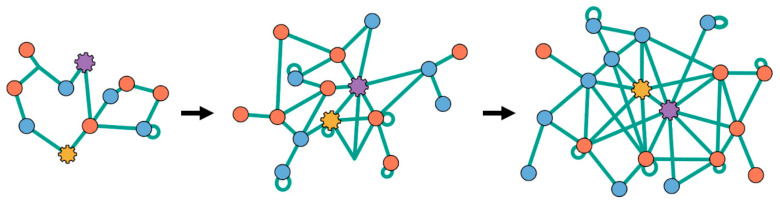
Example of a one-time evolution trajectory of a network. Initial network (left) is small and sparsely linked. Later networks have grown and changed through mutations discovered and lost, increasing the network’s complexity and size.

Figure 6 shows an example trajectory of an evolving PII network. It grows in nodes and edges. The network begins with only a few interactions. The average number of initial, randomly generated interactions in the starting network was 64 ± 6. In a network size of 50 different polymer types, split evenly between functional and information polymers, the average interaction per polymer was 2 ± 2. After 1,000,000 mutations, where some were fixed or lost, through processes resembling natural selection and genetic drift, the final network averaged 160 ± 16 total interactions. Here, each polymer type had on average 6 ± 3 interactions. Novel interactions between a polymer and either copy machine made up a fifth of all newly discovered interactions. Calculations regarding the number of interactions per polymer and the total initial and final network sizes were computed averages taken from the results of seven simulations.

The giant component of the network starts out sparse, with only a few interactions existing between polymer types. A component is defined as a group of nodes which are connected either indirectly or directly. Therefore, we define the giant component as the network component with the larger proportion of polymer types in it [43]. At time t = 0, when no mutations have been introduced into the system, a protocell’s growth rate is dictated solely by the chain elongation processes that occur from foldamer catalysis. To reflect this, our initial networks did not contain any copy machine subgraphs. Networks also started with a slight degree of fragmentation. Some polymer types were not connected with the giant component either through direct or indirect interaction. These polymer types became connected later in evolution as new interactions were discovered.

Mutations and selection result in an increase of network complexity. Interactions that are growth-rate neutral are observed most frequently. Protein copier and xNA copiers also discover fruitful interactions. The completion of a translation or transcription subgraphs provides substantial growth rate increases. Networks continue to grow in size as nonconnected polymers discover interactions with the giant component.

### 3.3. Bootstrap Model Network Topologies Resemble Today’s PPI Networks

**Figure 7:**
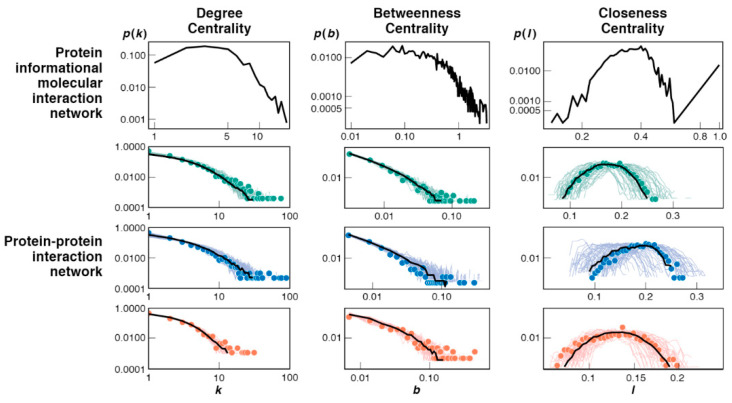
Topological node distributions of the bootstrap model networks resemble today’s cell PPI networks. (Top row) Predicted from this bootstrap model for protein–informational interactions (PPI). (Bottom) Measured protein–protein interaction (PPI) networks of humans (green), yeast (blue) and fruit flies (red); adapted with permission from [42], © 2012, Peterson et al. Since the size of the present simulation is far from a real-world PPI, direct comparison cannot be drawn; however the general behavior of these topological features shows that the present bootstrap mechanism gives a plausible evolutionary route to current cellular networks; see text.

The bootstrap model predicts how simple initial networks grow into more complex structures later in evolution. The structure and complexities of networks can be characterized by their topological features. Three are considered here: degree centrality, betweenness centrality and closeness centrality. The mathematical definition for all three centralities can be found in Appendix B, either written in text or shown in Equations (A7) and (A8). Figure 7 shows that these features predicted from the bootstrap model resemble the corresponding features of protein–protein interaction networks in present-day cells [42]. This comparison is made by comparing the present model’s topological features in a dynamic setting to those of static, fully evolved and simulated PPI network topologies. An important distinction we make here is that while the size of our PII network does not allow for a direct one-to-one comparison with known PPI networks, irrespective of that, the observed topologies of each share similar dependencies.

Here are the interpretations. First, if networks had many hubs—like many big cities in a traffic network—then the degree centrality plot would show large *p*(*k*) values at large values of *k* on these figures. However, that is not the case either from the bootstrap model or the experimental PPI data. Most proteins are connected to relatively few other proteins. There are very few hubs; they are mostly copy machines. Second, the between centrality reflects the number of bridges in the network. These are situations in which one molecule is a go-between linking two other molecules. In both the model and the PPI data, few molecules are bridging any two other molecules. In the model, the bridging molecules are largely the copy machines. Third, the closeness centrality shows the number of molecules that are either highly centralized (close to many other proteins) or highly decentralized, far away from other proteins. It measures the extent to which a molecule can interact with all other molecules in the network. The peaks in these plots indicate that most molecules are neither particularly isolated from others, nor crowded together with others. Our copy machines have high closeness values because of their hub-like nature. Direct interactions lead to many indirect interactions. A given molecule can make interactions with all other molecules without specificity in this model. Molecules that are nodal neighbors to the copy machines drive copy machines to become more centralized when they discover interactions to periphery molecules in the network.

Lastly, we briefly comment on the nature of other types of interactions networks, mainly DNA–protein networks, and RNA–protein networks. For brevity, we classify these as xNA–protein networks. At present, sufficient data is not available to compare xNA–protein network topology with the PII network. While recent efforts have been made to elucidate xNA–protein interactions [44,45,46,47], the databases housing the data do not provide graphical construction of the full interactome. Over the last decade, there has been a standardization in the data format and quality of PPI network data, but such standards do not exist for xNA–protein networks. Nevertheless, we are aware of a topological analysis on one graphical dataset [48] of a noncoding RNA–protein interaction network in yeast, but it only includes a small subset of interactions and not the full topological comparisons we seek here.

## 4. Conclusions

We develop here the bootstrap model for how proteins and nucleic acids might have evolved fruitful relationships in the origins of life. It is based on premises that we regard as plausible physical chemistry and maximal initial randomness. It supposes that xNA and protein molecules occupy vesicles. Since life requires nonequilibrium, our NEQ premise is the availability of persistent random short-chain syntheses of both polymers. Since life cannot exist without some form of survival-of-the-fittest propagation dynamic, we assume protocell colloids grow from the growing chains inside, and split, converting cell growth rates to cell populations. We suppose that the peptides are hydrophobic–polar (HP) polymers, and accept the previously elucidated foldamer hypothesis, wherein short HP peptides collapse hydrophobically in water, expose hydrophobic binding sites, and could, in principle, accelerate chain elongations with primitive ribosome-like and polymerase-like functionality. Random mutations can lead to growth advantages, spontaneous propagation and biochemical networks that have growing complexity. The biochemical network topologies predicted by the bootstrap model resemble those of today’s PPI networks in living cells.

## Data Availability

The computational code used for this model is available from the corresponding author on reasonable request.

## Appendix A

Here we give an approximate solution to the growth rate Equations (8) and (9). The full solution of these coupled differential equations is not readily obtainable, at least by us. However, it is possible to solve them for each single graph of the types in Figure 2 and we can then extrapolate the results in the case of many graphs of such type.

For a single graph of the functional type, the amount of protein *p* would be given by the following:

(A1) $$n_{p}(t)=n_{p}^{0}\exp(\int_{0}^{t}F\left(t\prime \right)dt\prime )+\frac{k_{F}}{L_{p}}t,$$

where $F\left(t\right)=\alpha \left(n_{r}^{0}m_{\mu }^{0}+\left(k_{F}m_{\mu }^{0}+k_{I}n_{r}^{0}\right)t+k_{F}k_{I}t^{2}\right)$. Now we assume that the splitting time $\tau_{s}$ is small enough that a first order approximation is valid. The result can be shown to be:

(A2) $$\tau_{s}\approx \frac{T_{s}}{1+\alpha \kappa_{p}n_{r}^{0}m_{\mu }^{0}},$$

(A3) $$\kappa_{p}=\frac{v_{F}n_{p}^{0}L_{p}}{v_{F}k_{F}N+v_{I}k_{I}M}.$$

Since the second term in the numerator $\alpha \kappa_{p}n_{r}^{0}m_{\mu }^{0}>0$ we see how the new splitting time is always smaller than the splitting time in absence of such machine $T_{s}$. This testifies that a single machine of this type is sufficient to boost the growth rate and hence the fitness of the protocell. We now extrapolate this result, assuming that this linear approximation holds on this case as well (true for sufficient small times) and we obtain the following expression for the growth rate of the entire system:

(A4) $$r\approx r_{0}+\sum_{p\mu r}A_{pr}A_{r\mu }\frac{\alpha r_{0}L_{p}}{k_{F}N+k_{I}{Mv}_{I}/v_{F}}n_{P}^{0}m_{\mu }^{0}n_{r}^{0},$$

where $\sum_{p\mu r}A_{pr}A_{r\mu }$ indicates the sum over all graphs of the functional type. Similarly for the xNA copiers, we can obtain an expression for the modified growth rate by solving the corresponding differential equation for a single graph and then extrapolating to an arbitrary number of graphs. The growth rate r summed over all possible graphs is:

(A5) $$r\approx r_{0}+\sum_{p\mu r}A_{pr}A_{r\mu }\frac{\alpha r_{0}L_{p}}{k_{F}N+k_{I}{Mv}_{I}/v_{F}}n_{P}^{0}m_{\mu }^{0}n_{r}^{0}\;\;\to +\frac{v_{I}}{v_{0}}\sum_{pv}A_{vv}A_{vp}\beta L_{v}{\left(m_{v}^{0}\right)}^{2}n_{p}^{0},$$

(A6) $$\equiv r_{0}+\Delta r_{R}+\Delta r_{C},$$

where $\sum_{pv}A_{vv}A_{vp}$ indexes all graphs of the xNA type. A convenient definition of fitness is the logarithm of the growth rate. Hence the rate can be decomposed into a component due to protein copiers, $\Delta r_{R}$ and a component due to xNA copiers, $\Delta r_{C}$.

## Appendix B

The degree centrality is the measure of links, k coming off a given node. The betweenness centrality is calculated using:

(A7) $$b=\sum \frac{\sigma_{st}(v)}{\sigma_{st}},$$

where $\sigma_{st}$ represents the totality of shortest path routes extending from arbitrary node *s* to node *t*. The term of $\sigma_{st}\left(v\right)$ represents the number of paths which pass through vertex *v*. The closeness centrality is calculated using:

(A8) $$l=\frac{N-1}{\sum_{s}d(t,s)},$$

where s≠t, *d*(*t*,*s*) is the length of the shortest path between nodes *t* and *s* in the network and *N* is the total number of nodes.

Reported graphs for degree, betweenness and closeness represent the dynamic topologies for the network as it evolves over time. Topological values are recorded for a network every 10,000 mutations. These values are compiled at the end of the simulation and a probability distribution for each topological feature is generated. The generated graphs are the median values of the probability distribution for each given value of k, b and l across all simulations.

Reported closeness values of l=0 are functions of randomized network generation and fragmentation. Occassionally some nodes in the graph at t=0 form a subgraph that is disconnected from the giant component. Due to the very small size of the disconnected subgraph, nodes will report high values for l because a centralized node might be connected directly to every node in the outlying subgraph.

## Appendix C

The magnitude of the reference growth rate was dependent on both static and dynamic parameters. Static parameters were not changed between simulations. These parameters included: *N*, *M*, $v_{F}$, $v_{I}$, $k_{F}$, and $k_{I}$. The preset values for each of these parameters can be seen in Table A1.

**Table A1:** Static parameters for the reference growth rate.

	*N*	*M*	$v_{F}$	$v_{I}$	$k_{F}$	$k_{I}$
Static Presets	25	25	0.143	0.303	10	10

Total polymer types for proteins and xNAs were limited to 25. This preset was selected as it offered good predictive power in relation to computational simulation time. Average volumes were computed from published literature volumes [49,50] and converted from ${Å}^{3}$ to ${\mathrm{nm}}^{3}$ to ensure poper parametric scaling. We assume that the polymer elongation rate for proteins and xNAs are equivalent, regardless of the mechanism behind polymerization. The preset for elongation rates of proteins and xNAs had been kept at 10 to keep the reference growth rate sufficiently small.

Dynamic parameters included: $n_{j}^{0}$, $L_{j}$ $m_{\mu }^{0}$, and $L_{\mu }$. These parameters were reset and reassigned at the beginning of each new protocell evolution trajectory. Lengths were assigned to each type of protein and xNA molecule using an exponential length distribution function adapted from the modeled data in the foldamer hypothesis [13]:

(A9) $$\;d\left(l\right)=0.067e^{-0.106x},$$

where *x* were chain lengths ranging from 10 monomeric units to 50 monomeric units. Subsequently, a list of 1000 pseudorandom variates were generated from the distribution and randomly assigned to each type of protein and xNA molecule in the model. The initial population size for each protein type and xNA type in the model were calculated using:

(A10) $$n_{j}^{0}=\frac{0.067e^{-0.106\left(L_{j}\right)}}{0.067e^{-0.106\left(length\;of\;shortest\;protein\right)}}\left(n_{j}^{0}\;of\;shortest\;protein\right),$$

(A11) $$m_{\mu }^{0}=\frac{0.067e^{-0.106\left(L_{\mu }\right)}}{0.067e^{-0.106\left(length\;of\;shortest\;xNA\right)}}\left(m_{\mu }^{0}\;of\;shortest\;xNA\right).$$

The initial population sizes of the shortest length protein type and xNA type were set to 1000 for every simulation. Lengths of $L_{j}$ and $L_{\mu }$ in Equations (A10) and (A11) corresponded to the length of the biomolecule for which the initial population size was being calculated for.
